# The biological interactions between kynurenine and AhR in melanocytes: in vitro studies

**DOI:** 10.1007/s00726-023-03279-0

**Published:** 2023-05-28

**Authors:** Katarzyna Walczak, Karolina Szalast, Dorota Krasowska

**Affiliations:** 1 Laboratory for Immunology of Skin Diseases, Chair and Department of Dermatology, Venereology and Paediatric Dermatology, Medical University of Lublin, Radziwillowska 11, 20-080 Lublin, Poland; 2 Department of Pharmacology, Chair of Pharmacology and Biology, Medical University of Lublin, Radziwillowska 11, 20-080 Lublin, Poland; 3 Chair and Department of Dermatology, Venereology and Paediatric Dermatology, Medical University of Lublin, Staszica 11Ł, 20-081 Lublin, Poland

**Keywords:** Kynurenine, Melanocytes, AhR, Cyclin D1, CDK4, Proliferation

## Abstract

Kynurenine (KYN), a tryptophan metabolite, is endogenously produced by the skin cells and is present in human sweat. The aim of this study was to determine the molecular mechanism of the antiproliferative activity of KYN on human epidermal melanocytes. KYN significantly inhibited the metabolic activity of HEMa cells by decreasing cyclin D1 and cyclin-dependent kinase 4 (CDK4) levels via the aryl hydrocarbon receptor (AhR) pathway. The results suggested that KYN might be involved in the regulation of physiological and pathological processes mediated by melanocytes.

## Introduction

Kynurenine (KYN), a tryptophan metabolite, is a key element of the kynurenine pathway. KYN is present in human biological fluids and tissues (Gál and Sherman 1980; Joseph 1978; Fujigaki et al. 1998; Widner et al. 1997; Mergola et al. 2018). Previous studies have revealed that KYN is involved in various biological processes, including immune response and modulation of neuronal function (Huang et al. 2020); however, the role of this tryptophan metabolite in physiological and pathological processes within the skin has not been fully elucidated. Sheipouri et al. reported that KYN was endogenously produced by human skin fibroblasts and keratinocytes and its concentration increased in response to UVB or interferon γ (IFN-γ) stimulation (Sheipouri et al. 2015). Moreover, KYN inhibited melanogenesis induced by tyrosine and NH_4_Cl (Ferreira Branquinho et al. 2022), but still, the direct effect of this tryptophan metabolite on melanocytes has not been fully revealed. Recently, we reported that KYN at a concentration of 5 mM significantly inhibited DNA synthesis in melanocytes (Walczak et al. 2020a), but the molecular mechanism of this interaction has not been studied.

Importantly, KYN is an endogenous ligand of aryl hydrocarbon receptor (AhR) (Opitz et al. 2011; DiNatale et al. 2010), which is mainly expressed in barrier tissues (Larigot et al. 2018). AhR plays an important role in physiological and pathological processes within the skin, including proliferation, differentiation, adhesion, migration, metabolism, pigmentation, cell signaling, and also cancer initiation and progression (Szelest et al. 2021; Fernández-Gallego et al. 2021).

In this study, we focused on the molecular mechanisms of the antiproliferative activity of KYN in human epidermal melanocytes. Additionally, the aim of this study was to determine the potential interactions between KYN and AhR in melanocytes.

## Materials and methods

### Cell culture

Normal human adult primary epidermal melanocytes (HEMa), obtained from American Type Culture Collection (PCS-200–013; ATCC; Manassas, VA, USA), were cultured in Dermal Cell Basal Medium supplemented with Adult Melanocyte Growth Kit (ATCC; Manassas, VA, USA). Cells were maintained in a humidified atmosphere of 95% air and 5% CO_2_ at 37 °C.

### MTT assay

HEMa cells were plated at a density of 4 × 10^4^ cells/mL in 96-well plates (Nunc, Roskilde, Denmark). Next day, the cells were exposed to serial dilutions of KYN (10^−9^, 10^−6^, 10^−3^, 1, 5 mM) or fresh cell culture medium (control, C) for 24 h or 96 h in standard conditions. L-KYN was dissolved in culture medium. Metabolic activity was assessed using the 3-(4,5-dimethylthiazol-2-yl)-2,5-diphenyl-tetrazolium bromide (MTT) assay previously described in Walczak et al. (2021). Briefly, MTT is reduced to water-insoluble formazan by metabolically active cells. Moreover, the number of cells in every well was counted.

### *AHR* silencing—siRNA

HEMa cells were plated on 6-well plates at a density of 5 × 10^4^ cells/mL the day before transfection with *AHR* siRNA (Assay ID  s1198; Thermo Fisher Scientific, Carlsbad, CA, USA ). Transfection was performed using Lipofectamine RNAiMAX (Thermo Fisher Scientific, Carlsbad, CA, USA) according to the manufacturer’s protocol. The *AHR* silencing was confirmed by RT-PCR. Negative controls showed no effect of reagents on *AHR* expression in HEMa cells (data not shown).

### Western blotting

HEMa cells were exposed to serial dilutions of KYN (10^−9^, 10^−6^, 10^−3^, 1, 5 mM) or fresh cell culture medium (control, C) for 24 h in standard conditions. The protein level of AhR, cyclin D1, and cyclin-dependent kinase 4 (CDK4) was measured by western blot procedure described in detail in Walczak et al. (2020b). Anti-cyclin D1, anti-CDK4, anti-AhR, and anti-β-actin primary antibodies (1:1000; Cell Signaling Technology, Danvers, MA, USA) and the secondary antibodies coupled to horseradish peroxidase (1:2000; Cell Signaling Technology, Danvers, MA, USA) were used in the procedure.

### Real-time PCR

HEMa cells were exposed to KYN 1 mM or fresh cell culture medium (control, C) for 24 h in standard conditions. Total RNA, isolated by High Pure RNA Isolation Kit (Roche Diagnostics GmbH, Penzberg, Germany), was reverse-transcribed using High Capacity cDNA Reverse Transcription Kit (Applied Biosystems, Foster City, CA, USA) according to the manufacturer’s protocol. TaqMan Gene Expression Assays for *AHR* (ID: Hs00169233_m1), *CCND1* (ID: Hs00765553_m1), *CDK4* (ID: Hs01565683_g1) and for *ACTB* (ID: Hs99999903_m1) (Thermo Fisher Scientific, Waltham, MA, USA), and TaqMan Fast Universal PCR MasterMix (Thermo Fisher Scientific, Waltham, MA, USA) were used in real-time PCR procedure as previously described in Langner et al. (2015).

### Fluorescent immunostaining

HEMa cells were exposed to KYN 1 mM or culture medium (control, C) for 24 h in standard conditions. Then, cells were treated according to the protocol described in Walczak et al. (2020b). Anti-AhR primary antibody (1:100; Cell Signaling Technology, Danvers, MA, USA) and secondary antibodies conjugated with fluorescein isothiocyanate (FITC) (1:100) (Sigma Aldrich, St. Louis, MO, USA) were used in the procedure. Cell nuclei were stained with DraQ5 (Cell Signaling Technology, Danvers, MA, USA).

### Data analysis

The data were shown as the mean value ± standard error of the mean (SEM). The results were statistically analyzed by GraphPad Prism 8 software (GraphPad Software, Inc., La Jolla, CA, USA) using one-way ANOVA with Tukey post hoc test or unpaired *t* test (*p* < 0.05). Western blots and RT-PCR were performed in triplicate. Western blots were quantified densitometrically by NIH ImageJ software (Wayne Rasband, Bethesda, MD, USA). The data were normalized to β-actin and presented as a relative value of control. RT-PCR results were normalized to *ACTB* expression (an endogenous control). The results represent a relative expression calculated by the RQ = 2^−ΔΔ*C*t^ formula.

## Results

**Fig. 1:**
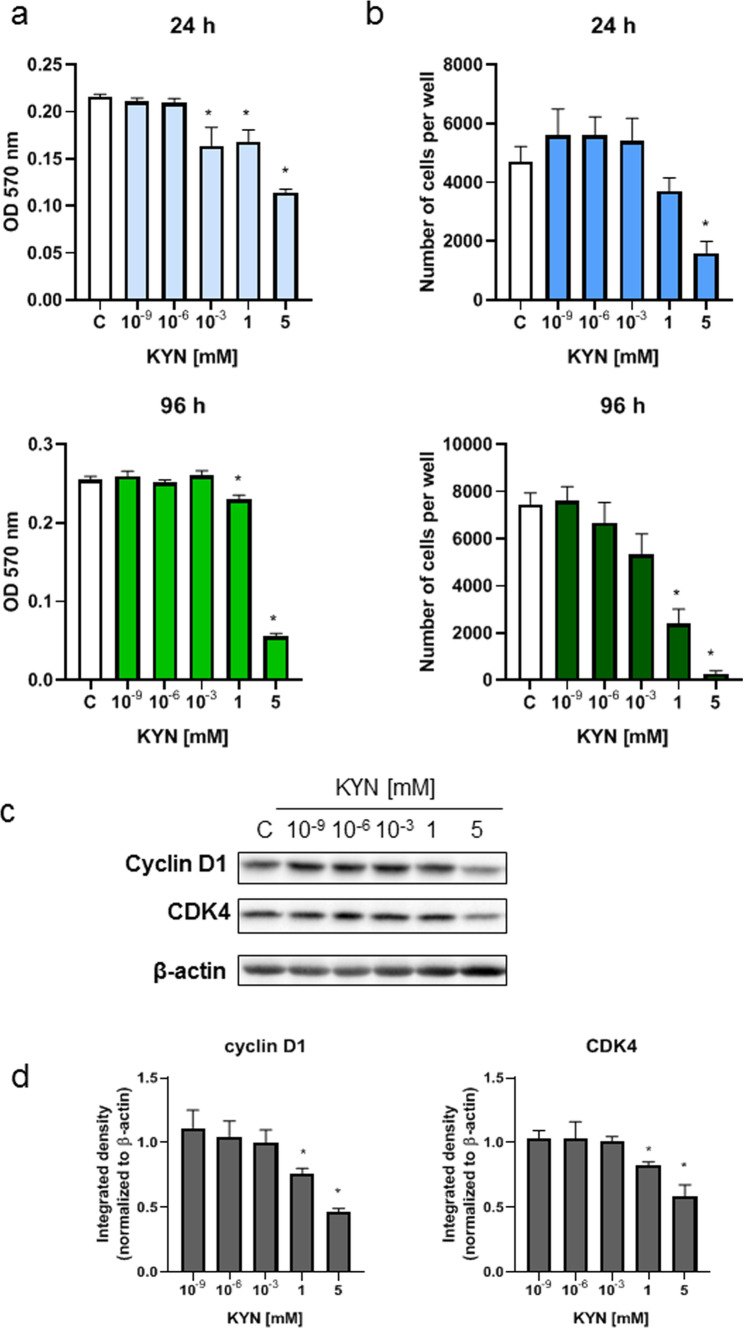
The effect of KYN on the proliferation and metabolic activity of melanocytes

KYN inhibited the proliferation and metabolic activity of HEMa cells in a dose-dependent manner (Fig. 1a, b). However, the strongest effect was observed in HEMa cells exposed to millimolar concentrations of KYN. Western blot analysis revealed that antiproliferative properties of KYN resulted from the inhibitory activity against cyclin D1 and CDK4 in HEMa cells (Fig. 1c, d).

HEMa cells were exposed to culture medium (control, C) or serial dilutions of KYN (10^−9^, 10^−6^, 10^−3^, 1, 5 mM) for 24 h or 96 h. Metabolic activity and proliferation were determined by MTT assay (**a**) and cell counting (**b**), respectively. Data were shown as the mean value ± SEM (biological replicates, *N* ≥ 6). Values significant (*) in comparison to control with *p* < 0.05 (one-way ANOVA, Tukey post hoc test). **c** The effect of KYN on the protein level of selected cell cycle regulators in melanocytes. The protein level of cyclin D1 and CDK4 was determined in HEMa cells by western blotting after 24 h incubation with the tested compound. Western blots showed the most representative one of the series of repetitions (*N* = 3). **d** Graphs presented the densitometric analysis of western blots (the data were normalized relative to β-actin). Values significant (*) in comparison to control (*C* = 1) with *p* < 0.05 (unpaired *t* test).

**Fig. 2:**
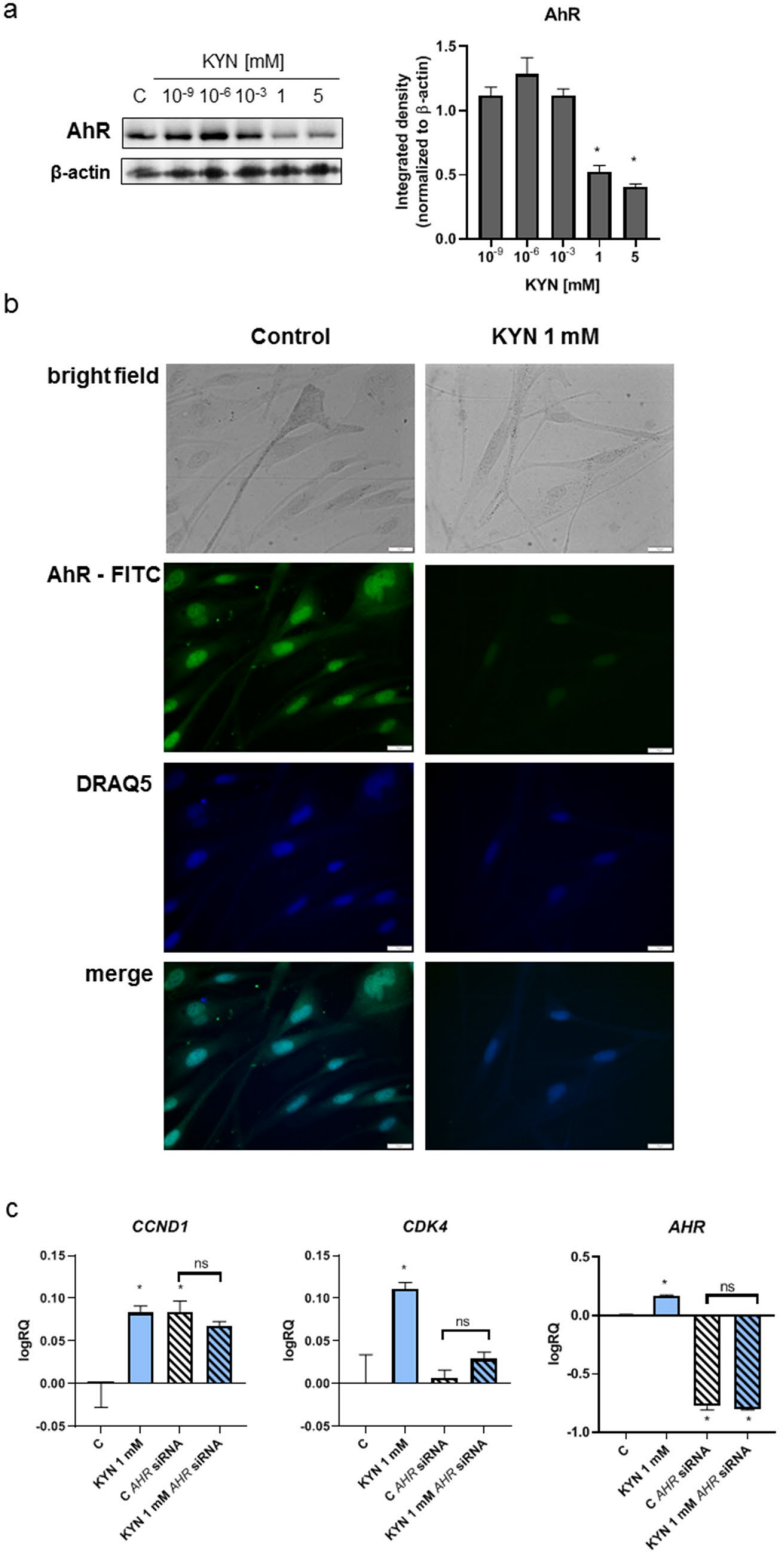
**a** The effect of KYN on AhR level in melanocytes. HEMa cells were exposed to culture medium (control, C) or serial dilutions of KYN (10^−9^, 10^−6^, 10^−3^, 1, 5 mM) for 24 h. Western blots showed the most representative one of the series of repetitions (*N* = 3). Graphs presented the densitometric analysis of western blots (the data were normalized relative to β-actin). Values significant (*) in comparison to control (*C* = 1) with *p* < 0.05 (unpaired *t* test). **b** The effect of KYN on the cellular localization of AhR in melanocytes. Immunofluorescent staining of AhR in HEMa cells treated with KYN 1 mM for 24 h (control; not treated). Cell nuclei were detected by cell-permeable fluorescent DNA dye DraQ5. Magnification 40 × . **c** The effect of KYN on gene expression of *CCND1*, *CDK4*, and *AHR* in melanocytes in standard conditions and after *AHR* silencing. HEMa cells were exposed to KYN 1 mM for 24 h (control, C; not treated). A similar experiment was performed in HEMa cells with silenced *AHR* gene (*AHR* siRNA). The gene expression data was obtained by RT-PCR (technical replicates, *N* = 3), normalized to *ACTB* expression, and shown as logRQ. Values significant (*) in comparison to control with *p* < 0.05 (unpaired *t* test)

KYN is considered an endogenous AhR ligand (Opitz et al. 2011). Therefore, we decided to check whether KYN might affect the protein level and gene expression of this receptor. Western blot analysis revealed that KYN in millimolar concentrations significantly inhibited the protein level of AhR in HEMa cells (Fig. 2a). Immunofluorescent staining confirmed decreased protein level of AhR in cells exposed to tested tryptophan metabolite (Fig. 2b). Importantly, nuclear translocation of AhR was not observed.

Previous results confirmed the involvement of KYN in cyclin D1 and CDK4 protein expression (Fig. 1c, d) and endogenous regulation of AhR (Fig. 2a); thus, the potential interaction between AhR and cell cycle regulation was studied. Interestingly, KYN exerted an opposite effect on *CCND1*, *CDK4,* and *AHR* gene expression in comparison to their protein level (Fig. 2c). KYN at a concentration of 1 mM significantly increased the gene expression of *CCND1*, *CDK4,* and *AHR*. Importantly, this effect was not observed in HEMa cells with silenced *AHR* gene (Fig. 2c).

## Discussion

In this study for the first time, we reported the potential molecular mechanism of KYN activity on human epidermal melanocytes. KYN, a tryptophan metabolite and a key element of the kynurenine pathway, affected cell cycle regulation in HEMa cells via the AhR pathway. Although further studies are necessary, the results suggested that KYN, considered an endogenous AhR ligand (Opitz et al. 2011), might be involved in the regulation of physiological processes within the skin.

Previous studies confirmed the presence of KYN in human sweat; however, the exact concentration and origin of KYN in sweat are still discussed (Saran et al. 2021; Jankovskaja et al. 2022). KYN is produced endogenously by skin fibroblasts (0.24 ± 0.013 μM) and keratinocytes (0.51 ± 0.027 μM) in vitro (Sheipouri et al. 2015). Additionally, the stimulation by UVB or IFN-γ led to over ten times higher KYN production in fibroblasts (Sheipouri et al. 2015). Moreover, previous studies revealed that the skin microbiome might participate in tryptophan metabolism leading to KYN production or changing the balance of enzymatic reactions of the kynurenine pathway and indirectly modifying KYN concentration within the skin (Guenin-Macé et al. 2020; Yu et al. 2019). KYN is also present in honey, soybean, sesame, pumpkin, and spirulina extracts, which may be found in various cosmetics used in skincare and body treatments (Soto et al. 2011; Vitalini et al. 2020). Although the significant effect of pico- and nanomolar concentrations of KYN was not revealed in this study, it should be underlined that KYN concentration on the surface of the skin may significantly increase taking into consideration sweat evaporation. Thus, despite high concentrations of KYN exerting a biological effect on human epidermal melanocytes, the results are biologically important. Saran et al. reported that KYN is present in human sweat in an average amount of 73.93 fmol/µg Na with the average sodium content of the sample equal to 183.77 µg (Saran et al. 2021). In the other studies, collected KYN in sweat did not exceed 20 ± 10 pmol/cm^2^ (Jankovskaja et al. 2022), but at this stage of studies, it is not clear whether this amount resulted from limitations of sample collection and detection, or KYN is transformed into other tryptophan derivatives under visible or UV radiation, or microbiome transformation.

Previous studies reported an inhibitory potential of KYN against melanocytes (Walczak et al. 2020a). KYN at a concentration of 5 mM significantly inhibited DNA synthesis, but did not induce cytotoxicity in HEMa cells (Walczak et al. 2020a). In this study, we confirmed that KYN inhibited not only DNA synthesis, but also decreased even more potent metabolic activity of HEMa cells determined by the MTT assay (Fig. 1a). Importantly, MTT is transformed only by metabolically active cells. Therefore, the MTT assay determines the metabolic activity of cells and, indirectly, cell viability (Berridge et al. 2005; Ghasemi et al. 2021). Ferreira Branquinho et al. reported that KYN decreased the level of melanin in melanocyte and keratinocyte co-cultures inhibiting the expression of tyrosinase (Ferreira Branquinho et al. 2022). However, our study confirmed that KYN activity toward human melanocytes was more complex. KYN inhibited not only melanogenesis, but also was involved in the proliferation, cell cycle regulation and metabolic activity of melanocytes via the AhR signaling pathway.

KYN affected cell cycle regulation decreasing the protein level of cyclin D1 and CDK4 (Fig. 1c). A similar molecular mechanism of KYN activity was previously revealed in melanoma A375 and RPMI-7951 cells (Walczak et al. 2020a). Moreover, bioinformatics analysis confirmed the interactions between the kynurenine pathway, cyclin D1, and CDK4 in melanoma (Wang et al. 2022). Interestingly, the gene expression data suggested that the biological activity of KYN was the result of its influence on gene expression and protein products. It cannot be excluded that melanocytes compensated the protein degradation by enhanced expression of *CCND1* and *CDK4* (Fig. 2c). A similar effect was observed in AhR protein level and *AHR* gene expression (Fig. 2a, c). KYN at millimolar concentrations significantly decreased AhR level in HEMa cells, whereas *AHR* gene expression was enhanced. Although the decrease of AhR protein level was also previously reported in human melanoma A375 and RPMI-7951 cells exposed to KYN, this tryptophan metabolite did not affect gene expression of *AHR* in melanoma cell lines (Walczak et al. 2020a). Unfortunately, the regulation of *AHR* expression by its endogenous ligands in melanocytes has not been studied so far. The majority of studies reported increased protein level of AhR in immune cells exposed to KYN (Liu et al. 2018; Zhang et al. 2021; Manni et al. 2020). On the other hand, the opposite effect of KYN on AhR protein level was previously observed in HepG2 cells (Che and Dai 2019). Importantly, Kaiser et al. proposed the functional regulatory network between AhR, the kynurenine pathway, the NF-κB signaling, and the amino acid transporter SLC7A5 (Kaiser et al. 2020). Our results suggested that there could be different self-regulation mechanisms or different biological interactions between KYN and AhR in normal and cancer cells within the skin. Importantly, KYN decreased the level of AhR in HEMa cells but it did not lead to nuclear translocation of AhR, which suggested that KYN in higher concentrations might be a negative regulator of AhR in melanocytes preventing overactivation of the AhR signaling. Previous studies reported that AhR activation by 6-formylindolo(3,2-b)carbazole (FICZ) might promote posttranslational degradation (Mengoni et al. 2020). However, further studies are necessary to confirm this hypothesis.

In this study, we found the functional interaction between KYN, AhR, and cell cycle regulation in melanocytes. KYN at a concentration of 1 mM enhanced the expression of *CCND1* and *CDK4* in HEMa cells but its activity was lost in *AHR*-silenced cells. Importantly, *AHR* silencing led to an increase in *CCND1* expression, which confirmed the involvement of the AhR signaling in the proliferation of melanocytes.

In conclusion, KYN, an endogenous tryptophan metabolite, might be involved in the proliferation and cell cycle regulation of human epidermal melanocytes via the AhR signaling. It should be noted, that KYN has immunomodulatory properties through interaction with natural killer cells, dendritic cells, monocytes, macrophages, and T cells (Cervenka et al. 2017). In our experiments, we studied only the direct effect of this tryptophan metabolite on melanocytes in vitro. However, the biological activity of KYN in the skin is probably more complex including the direct interaction with the skin cells and modulation of the immune response. Taking into consideration the potential direct and indirect influence of KYN on the skin, it cannot be excluded that KYN may be involved in the pathogenesis of pigmentation disorders, including vitiligo or hyperpigmentation.

## Data Availability

Data sharing not applicable to this article as no datasets were generated or analyzed during the current study.

## Abbreviations

AHR: Aryl hydrocarbon receptor
ATCC: American Type Culture Collection
CDK: Cyclin-dependent kinase
FICZ: 6-Formylindolo(3,2-b)carbazole
FITC: Fluorescein isothiocyanate
HEMa: Human adult epidermal melanocytes
IFN-γ: Interferon γ
KYN: Kynurenine
MTT: 3-(4,5-Dimethylthiazol-2-yl)-2,5-diphenyltetrazolium bromide
NAD: Nicotinamide adenine dinucleotide
SDS: Sodium dodecyl sulfate
